# Availability of New Medicines in the US and Germany From 2004 to 2018

**DOI:** 10.1001/jamanetworkopen.2022.29231

**Published:** 2022-08-30

**Authors:** Katharina Blankart, Huseyin Naci, Amitabh Chandra

**Affiliations:** 1 Faculty of Economics and Business Administration, University of Duisburg-Essen, Essen, Germany; 2 London School of Economics, London, United Kingdom; 3 Harvard Kennedy School, Harvard University, Cambridge, Massachusetts

## Abstract

**Importance:**

Germany’s unique approach to coverage determination and pricing has ensured that effective medicines remain on the market, often at prices reduced through negotiation. However, less is known about trade-offs of this approach with regard to initial availability of medicines.

**Objective:**

To examine differences in the timing and scope of new medicines available in Germany and the US.

**Design, Setting, and Participants:**

This retrospective cohort study analyzed initial availability of new medicines approved by regulatory agencies in Germany and the US between January 1, 2004, and December 31, 2018, and followed up through December 31, 2019. Data analysis was conducted from January 1, 2020, to July 1, 2022. A total of 599 novel approvals were reviewed. Generic, biosimilar, vaccine, and combination medicines were excluded.

**Exposures:**

US Food and Drug Administration approvals were reviewed for therapies categorized as new molecular entities or new active ingredients. German approvals were reviewed from secondary administrative data of authorized medicines that determine availability in Germany, including data presented by the European Medicines Agency.

**Main Outcomes and Measures:**

Approvals were analyzed to determine the percentage of medicines approved and available in the US, Germany, or both countries and compare the times to reach the market.

**Results:**

Analysis of 599 new medicines demonstrated that fewer were available in Germany compared with the US (80% vs 92% of all potential therapies) and that the median difference in time to market was 4 months (95% CI, −44.40 to 44.76 months). Forty-nine medicines were approved in Germany but not in the US, 75% of which were rejected by the US Food and Drug Administration, were withdrawn, or had US equivalent agents.

**Conclusions and Relevance:**

In this cohort study, fewer new medicines were available in Germany compared with the US between 2004 and 2018. In addition, drugs entered the German market later than in the US.

## Key Points

**Question:**

Is there a difference in the availability of new drugs in Germany and the US?

**Findings:**

In this cohort study comparing 599 medicine approvals in the US and Germany between 2004 and 2018, 92% of all new medicines approved in either country were available in the US, and 80% were available in Germany. The median approval time in Germany was extended by 4 months.

**Meaning:**

The findings of this study suggest that, compared with the US, fewer new medicines are available in Germany, and they enter the market at a later time.

## Teaser

This cohort study compares the medications approved in Germany and the US and differences in approval time.

## Introduction

United States policy makers are interested in understanding how other countries approach the challenge of high drug prices. One country that is often highlighted for its unique approach is Germany, which allows manufacturers to set unconstrained prices for medicines at launch but requires that all medicines undergo a systematic assessment of their effectiveness, permitting insurers to renegotiate prices after this assessment.^1^ This policy has guaranteed that almost 98% of effective medicines for specific indications are kept on the market and increased the likelihood 10-fold that medicines not deemed beneficial were withdrawn from the market.^2,3,4^ Moreover, for cancer drugs, price negotiations have resulted in prices being reduced by 24.5% 1 year after launch.^5^

Less known, however, is whether the number of medicines available in Germany is meaningfully smaller than what is available in the US. Earlier studies found that new drugs often enter the US market first.^6,7^ The US Food and Drug Administration (FDA) may also approve more medicines in part by relying on surrogate end points, but there is also evidence that the European Medicines Agency (EMA) uses similar end points.^8^ The recent decision to approve a controversial medicine for Alzheimer disease in the US but not in Europe, supports these views.^9,10^ Approvals by the EMA in combination with German requirements for market entry may be more restrictive than US approvals for some medicines but more permissive for others.^11^

We performed a retrospective cohort study of novel drugs that were available in both the US and Germany, medicines that were available in Germany but not in the US, and medicines that were available in the US but not in Germany. We evaluated the time it took medicines to reach the German market relative to the US market.

In the US, the FDA approves medicines, and commercial payers, which cover one-half of the US population, make their own coverage decisions. However, the process behind commercial insurers’ coverage determinations is opaque and variable across insurers; these determinations are not based on a transparent cost-effectiveness analysis of the type conducted by the Institute for Clinical Effectiveness Research. The 2 largest public payers in the US, Medicare and Medicaid, insure the other half of US citizens and are de facto required to cover most approved therapies (the Veterans Administration uses a formulary but is relatively small; it covers 9 million US citizens, 2.5% of the population) with some management tools in Medicaid.

In Germany, medicine availability reflects a 3-step process. First, for most new medicines, the EMA makes an approval decision for all European Union member states (this is the mandatory process for treatments for HIV and AIDS, oncology, diabetes, neurodegenerative disorders, autoimmune diseases, and viral diseases).^12^ After this approval, coverage decisions are triggered if manufacturers decide to enter the German market. Manufacturers can apply directly to the German authorities for approving medicines outside of the mandatory classes. Market entry triggers decision-making by the Federal Joint Committee about the drug’s health benefit, which is a nonstate self-governance body that includes payer, health care professional, and patient representatives (created by Arzneimittelmarkt-Neuordnungsgesetz [AMNOG] in 2011).^5^

Next, the Institute for Quality and Efficiency in Health Care (IQWiG), usually commissioned by the Federal Joint Committee, compares new therapies with standard of care—not placebo—for a given indication and makes a determination of coverage. The institute takes a holistic view of the evidence, including studies not judged by the FDA to be pivotal. As a result, the IQWiG can consider a variety of outcomes, including morbidity, mortality, quality of life, and patient experience, before making a decision. The IQWiG is funded through contributions from all German health insurance funds. Analysis conducted by the IQWiG does not apply to medicines for rare diseases with expected revenues below €50 million per year (US $57 million), which account for almost one-half of all approvals and often have high prices.

Relying on and augmenting the IQWiG analysis, the Federal Joint Committee determines whether the benefit from a medicine is commensurate with the current treatment standard. Depending on the Federal Joint Committee determination, the manufacturer can negotiate a price with insurers, price to match an equivalent medicine, or withdraw the medicine from the market. Before the introduction of AMNOG, health technology assessments were not required regularly to determine negotiated prices and were used in select reference pricing exercises; the pre-AMNOG environment resembled the US situation today.

## Methods

### Design

This was a retrospective cohort study of all 599 medicines approved in the US and Germany between January 1, 2004, and December 31, 2018, followed up until December 31, 2019. The initial year of analysis is 2004 because it is the year the EMA introduced the mandatory centralized authorization procedure for all new active ingredients approved in European Union Member States. Our focus was on the initial marketing authorization and related activities at the national level. We therefore did not consider subsequent activities that determine prescribing decisions by physicians and use by patients. We also did not consider any withdrawals after initial market entry. Data analysis was conducted from January 1, 2020, to July 1, 2022. This study conforms to the Strengthening the Reporting of Observational Studies in Epidemiology (STROBE) reporting guideline.

### Data Sources

To identify novel drug therapies, we used publicly available information from the FDA, the EMA, and data on market launches in Germany based on administrative data. For the US, we considered all medicines approved by the FDA, relying on documents provided through the Orange Book.^13^ We included 458 approvals designated as original submissions that were ever approved by the FDA and were categorized as a new molecular entity (type 1) or new active ingredient (type 2) to reflect the somewhat broader definition of a new active substance by European standards.^14^ For Germany, we extracted data on 462 novel drug therapies approved using an annual report of the German pharmaceutical market (Arzneiverordnungs-Report)^15^ that relies on administrative data provided by ABDATA-Pharma-Daten-Service.^16^ Approval is the first time a medicine was available for prescription in the German market, which may deviate from the date of market approval by the EMA or the German national marketing authorization agency because drug presentations need to be registered for marketing. For both the US and Germany, we also checked whether medicines have been available before the starting point of our observation based on the same data sources.

In addition, we collected data from the EMA based on public assessment reports.^17^ Within the European Union, medicines may be launched centrally by the EMA, which is the pathway for most new medicines, or by application to national regulators. To validate approval status and additional information, especially for drugs that were not launched in both the US and Germany, we accessed drugbank.com^18^ and Gelbe Liste^19^ for Germany.

### Identifying New Drug Approvals in the US and Germany

We identified 599 unique novel therapies that were launched in the US or German market. We mapped drugs by brand and active ingredient based on the international nonproprietary name. We verified whether the same active pharmaceutical ingredient is marketed under different brand names. We did not consider medicines that were subject to compassionate use or considered as investigational or experimental. We excluded vaccine, generic, and biosimilar agents because these are considered in separate approval processes by the FDA and outside the scope of the German AMNOG process.

### Identifying Availability of Medicines by Country

For each medicine, we captured the first date of market availability by month and year. As outcomes based on approval status, we checked whether the 599 medicines were ever available in the US, Germany, or both. For medicines that were approved in 1 of the 2 countries, we identified whether these agents were approved at an earlier time (ie, before 2004) or were never approved.

### Determining Availability of Medicines by Approval Status

Approvals were analyzed to determine the percentage of all new medicines available in the US, Germany, or both. We first calculated the percentage of medicines approved in the US and Germany as the percentage of all medicines approved.

For the subset of medicines that we identified to be approved in either the US or Germany, we calculated the difference in approval times in months, with the US approvals as the reference country. We computed the median (95% CI) time difference between approvals. Because the exact month of approval was missing in the German data for 2 medicines, we imputed the mean month (April).

For the subset of medicines that were approved in Germany but not the US, we classified these into 5 categories that are suggestive about potential reasons of nonapproval and 1 residual category. We classified orphan drug designation of medicines using information provided by the EMA for Germany and by the FDA based on the Orphan Drug Designations and Approvals database for the US. We classified small-molecule or biologic drug status based on the type of application process (new drug application compared with biologic application) and data from drugbank.com for medicines approved by the FDA. According to the labeling information, we noted whether medicines were indicated to treat cancer. We also noted whether they were subject to an early assessment of health benefit according to AMNOG in Germany after 2011 by their initial approval date. We classified whether the medicine previously received marketing authorization by the EMA or was suspended. Based on the FDA Orange Book, we determined the priority vs standard review designation.

### Statistical Analysis

The 95% significance threshold was determined using unpaired, 2-sided tests. All data were maintained and processed in SAS, version 9.4 (SAS Institute Inc) and analyzed in Microsoft Excel (Microsoft Corp) and SAS.

## Results

Table 1 describes our sample of medicines. In total, 599 medicines were approved in either Germany or the US between 2004 and 2018, representing the maximum number of new medicines theoretically available for prescription in these countries.

**Table 1.** Medicine Approvals in the United States and Germany, 2004-2018

Medicine approved in Germany	Medicine approved in the US, frequency (%)			
	Approved in US before 2004	Never approved in US	Approved in US in 2004-2018	Total
Approved in Germany before 2004	0	0	36 (6.01)	36 (6.01)
Never approved in Germany	0	0	123 (20.53)	123 (20.53)
Approved in Germany 2004-2018	73 (12.19)	49 (8.18)	318 (53.09)	440 (73.46)
Total	73 (12.19)	49 (8.18)	477 (79.63)	599 (100)

Of these potential medicines, 12% (n = 73) had already been approved in the US before 2004 (Germany approved these after 2004), and 80% (n = 477) gained approval in the US between 2004 and 2018. Therefore, 91.8% of potential medicines (477 + 73 of 599 medicines) were available in the US. In Germany, 36 (6%) of the 599 possible medicines had been approved before 2004, and 440 (74%) subsequently received approval. Therefore, 79.5% of potential medicines were available in Germany. When we compared time to availability in the US and Germany for the medicines that were approved in both countries, it took 4 months longer for the median therapy (95% CI, −44.40 to 44.76 months) to enter the German market, including the EMA review times. Forty-nine medicines were approved in Germany but not in the US, 75% of which were rejected by the US Food and Drug Administration, were withdrawn, or had US equivalent agents.

To understand whether the differences in medicine availability were clinically meaningful, we examined the medicines that were available in either Germany or the US but not in both countries (Table 2). Medicines available only in Germany are noted in Table 3.

**Table 2.** Medicines Approved in Germany or the US But Not in Both Countries, 2004-2018

Approval variable	Frequency (%)	
	Not approved in US but approved in Germany (n = 50)	Not approved in Germany but approved in US (n = 123)
Orphan drug designation^a^		
No	41 (82.00)	66 (53.66)
Yes	9 (18.00)	57 (46.34)
Type		
Biologic	8 (16.33)	24 (19.52)
Small molecule	40 (81.64)	99 (80.48)
Stem cell therapy	1 (2.04)	0
Cancer treatment		
No cancer	41 (83.67)	101 (82.11)
Cancer	8 (16.33)	22 (17.89)
Launched before or after AMNOG was implemented		
Before	24 (48.98)	25 (20.33)
After	25 (51.02)	98 (79.67)
EMA authorization status		
Not approved	21 (42.96)	93 (75.61)
Approved^b^	21 (42.86)	23 (18.70)
Withdrawn, refused, suspended	7 (14.29)	7 (5.69)
FDA review priority status		
Priority	NA	65 (52.85)
Standard	NA	58 (47.15)

Abbreviations: AMNOG, German Pharmaceutical Market Restructuring Act (Arzneimittelmarktneuordnungsgesetz); EMA, European Medicines Agency; FDA, Food and Drug Administration; NA, not applicable.

^a^ The EMA designation for German approvals or FDA designation for US approvals.

^b^ No approvals with accelerated approval pathways.

**Table 3.** Medicines Approved in Germany But Not in the US, 2004-2018

Medicine	Trade name	Indication	Comment
**Available in Germany without US equivalent at present (n = 6)**			
Carbetocin	Pabal	Prevention of uterus atony after cesarean delivery	None
Corneal epithelial cells	Holoclar	Limbal stem cell insufficiency in the eye	None
Levosimendan	Simdax	Severe chronic heart failure	No novel therapy according to AMNOG
Nabiximols	Sativex	Spasticity in multiple sclerosis	In FDA trials at time of writing
Velmanase alfa	Lamzede	Alpha-mannosidosis	In FDA trials at time of writing
Vinflunine	Javlor	Urothelial cancer	None
**Available in Germany but rejected by the FDA (n = 12)**			
Alipogene tiparvovec	Glybera	Familial lipoprotein lipase deficiency	Weak data and benefit is unlikely to be sustained
Ataluren	Translarna	Duchenne muscular dystrophy	Missed primary and secondary end points in multiple studies
Corifollitropin	Elonva	Controlled ovarian stimulation	Received complete response letter from FDA in 2014
Dapoxetine	Priligy	Premature ejaculation	Rejected by FDA
Idebenone	Raxone	Leber hereditary optic neuropathy	Failed phase 3 trial
Lumiracoxib	Prexige	Osteoarthritic pain	Rejected by FDA, no significant difference vs existing options
Mifamurtide	Mepact	Malignant osteosarcoma	Rejected by FDA
Padeliporfin	Tookad	Low-risk prostate carcinoma	Adcom rejected, stating that another drug is more effective without the side effects
Pixantrone	Pixuvri	Non-Hodgkin lymphoma	Rejected by FDA, failed 2 RCTs
Tivozanib	Fotivda	Renal cell carcinoma	FDA has rejected multiple times already for weak data
Vernakalant	Brinavess	Antiarrhythmia	Rejected 11-2 by FDA for poor risk-benefit profile
Ximelagatran	Exanta	Thromboembolism prophylaxis	Rejected by FDA for weak efficacy and safety, several anticoagulant alternatives available
**Available in Germany and subsequently withdrawn (n = 9)**			
Allogenic genetically modified t cells	Zalmoxis	Adjuvant therapy for haplo-identical hematopoietic stem cell transplantation	EMA conditional authorization withdrawn in 2019
Catumaxomab	Removab	Malignant ascites	Voluntarily withdrawn from the market
Efalizumab	Raptiva	Psoriasis vulgaris	None
Eptotermin alfa	Osigraft	Tibia fracture with pseudarthrosis	None
Gaxilose	LacTest	Lactose intolerance	Opted out of Germany after AMNOG assessment, never obtained EMA approval
Laropiprant with nicotinic acid	Tredaptive	Dyslipidemia	Shown to have no benefit, previously withdrawn from the EMA by Merck
Melagatran	Melagatran (AstraZeneca)	Thromboembolism prophylaxis	Withdrawn across the board for liver toxic effects
Rimonabant	Acomplia	Obesity	Withdrawn in 2009
Sitaxentan	Thelin	Pulmonary arterial hypertension	Removed from market in 2010; rejected by FDA
**Available in Germany and with US equivalents (n = 16)**			
Agomelatin	Valdoxan	Major depression	No major difference vs bupropion or mirtazapine
Bilastine	Bitosen	Allergic rhinoconjunctivitis, urticaria	Many options in US (some OTC, some prescription, most generic)
Ceftobiprol medocaril	Zevtera	Nosocomial pneumonia	Several generic options (including of the same class [cephalosporin])
Cholic acid	Orphacol	Congenital disorder of primary bile acid synthesis	Cholbam from Retrophin as their alternative
Delamanid	Deltyba	Multidrug-resistant tuberculosis	Pretomanid is FDA approved
Desfesoterodine	Tovedeso	Incontinence	No different than fesoterodine, which is FDA approved
Lipegfilgrastim	Lonquex	Neutropenia	Pegfilgrastim is approved
Nadifloxacin	Nadixa	Acne vulgaris	Other options available
Nomegestrol acetate	Zoely	Oral contraception	Other options available (most generic)
Nomegestrol acetate with estradiol	Naemis	Hormone replacement therapy in postmenopausal women	Other options available
Racecadotril	Tiorfan	Diarrhea in children	Other opioid options available
Rupatadine	Rupafin	Allergic rhinitis	Many options in US (some OTC, some prescription, most generic)
Tegafur, gimeracil, oteracil	Teysuno	Advanced gastric cancer	None
Tianeptine	Tianeurax	Depression	None
Vildagliptin	Galvus	Type 2 diabetes	Other options available
Zofenopril	Bifril, Zofenil	Hypertension, acute myocardial infarction	None
**Approved by the FDA after 2018 or under evaluation (n = 6)**			
Ferric maltol	Accrufer, Feraccru	Iron deficiency	None
Etoricoxib	Arcoxia	Rheumatoid arthritis	Additional safety and efficacy data required
Landiolol	Rapibloc	Short-term control of tachyarrhythmias	None
Nabiximols	Sativex	Multiple sclerosis	None
Opicapone	Ongentys	Parkinson disease	None
Rurioctocog alfa pegol	Adynovi	Hemophilia A	None

Abbreviations: AMNOG, German Pharmaceutical Market Restructuring Act (Arzneimittelmarktneuordnungsgesetz); EMA, European Medicines Agency; FDA, Food and Drug Administration; OTC, over the counter; RCTs, randomized clinical trials.

Many of the 123 medicines that were available in the US but not in Germany received FDA approval in the most recent year of our study period and therefore could still be approved in the European Union or in Germany (eTable in the Supplement). Ninety-four medicines were not approved by the EMA during the study period, 18 were approved by the FDA in 2018 and could still be approved, and 7 were refused or withdrawn by the EMA. Four medicines with approval, of which 2 had no substitutes, were subsequently approved outside the follow-up period.

## Discussion

In this study, we compared the availability of new medicines in the US and Germany. The German system—which relies on the EMA for most approvals—included fewer medicines available during the study period and had access to medicines slightly more slowly, likely due to duration of EMA review.^7^ Eighty percent of all potential medicines were available in Germany and 92% were available in the US. This analysis highlights 2 differences between the US and Germany—earlier approvals and more approvals in the US. We found that several medicines that were not ultimately approved by the FDA were available in Germany, and some were subsequently withdrawn by the European Union. Medicines with therapeutic substitutes were available on both markets. More research is needed to determine whether medicines that are available in the US but not in Germany offer added therapeutic benefits over existing alternatives.

The US offers access to a greater number of new medicines, with the approval process marginally faster by 4 months. The process does not seem to compromise safety for speed relative to the European Union and Germany—during our study period, the FDA did not approve several medicines that the European Union subsequently rejected. However, for Medicare and Medicaid, the de facto link between approval and coverage reduces the ability of commercial insurers to refuse coverage, thereby increasing prices. As it stands, the US approval system can lead to high drug prices and an increased likelihood that these medicines are prescribed without strong evidence of efficacy.^5^ Perhaps most importantly, 10% of US patients remain uninsured, and many insured patients still face significant administrative barriers and cost-sharing to access therapies, such as prior authorizations and step therapy protocols. Access in the US, therefore, is more complicated than regulatory approval. Once a medicine is available in Germany, access to prescription medicines is uniform and cost-sharing is modest—between €5 and €10 (US $5.08-$10.17, as of August 8, 2022)—and independent of patent protection status.

### Limitations

This study has limitations. The sample was limited to all medicines approved between 2004 and 2018 and followed up through 2019 such that our findings may not be generalized to medicines in other time periods or to agents that we did not study. In addition, we did not capture withdrawals from the European Union market that are not attributable to decisions made by German authorities.

## Conclusions

Our retrospective cohort study of medicines approved in the US and Germany reveals a variety of trade-offs that health systems may wish to consider. These trade-offs may be justifiable but will require more explicit exploration of forgone medicines in Germany, including time differences in market availability owing to reliance on European regulatory frameworks, and higher prices and cost-sharing in the US. Expanding this analysis to other countries, including Canada, the UK, and Japan, to understand the quality of regulatory science in each country would be a fruitful area for future research.

## Notes

Supplement. **eTable.** Medicines Approved in the US But Not in Germany
