# Supplementary material for: Availability of New Medicines in the US and Germany From 2004 to 2018
Source: JAMA Netw Open. 2022 Aug 30;5(8):e2229231. doi: 10.1001/jamanetworkopen.2022.29231 (PMC9428736; doi:10.1001/jamanetworkopen.2022.29231)
Supplement: Supplement. — eTable. Medicines Approved in the US But Not in Germany [file jamanetwopen-e2229231-s001.pdf]

Supplementary Online Content

Blankart K, Naci H, Chandra A. Availability of new medicines in the US and Germany from 2004 to 2018. *JAMA Netw Open*. 2022;5(8):e2229231. doi:10.1001/jamanetworkopen.2022.29231

eTable. Medicines Approved in the US But Not in Germanye

This supplementary material has been provided by the authors to give readers additional information about their work.

© 2022 Blankart K et al. *JAMA Network Open.*

Supplementary Table 1: Medicines Approved in the U.S. but not in Germany

**Panel A: Medicines available in U.S. without German equivalent at present**			
ANGIOTENSIN II ACETATE	GIAPREZA	Increasing blood pressure in adults with septic or other distributive shock.	Approved outside study period, no added benefit identified according to G-BA's appraisal
NERATINIB MALEATE	NERLYNX	Early stage HER2-overexpressed/amplified breast cancer	Became available outside study period
**Panel B: Medicines available in U.S. but unauthorized / rejected by the EMA, including medicines approved by the FDA in 2018 and not (yet) approved by EMA in 2019**			
ACALABRUTINIB	CALQUENCE	treatment of adult patients with Mantle Cell Lymphoma who have received at least one prior therapy.	Not approved by EMA, orphan designation
ALOGLIPTIN BENZOATE	NESINA	Adjunct to diet and exercise to improve glycemic control in adults with Type 2 diabetes mellitus.	Not approved by EMA
ALVIMOPAN	ENTEREG	Accelerate the time to upper and lower gastrointestinal recovery following partial large or small bowel resection surgery with primary anastomosis	Not approved by EMA
AMMONIA N-13	AMMONIA N 13	For diagnostic Positron Emission Tomography imaging of the myocardium under rest or pharmacologic stress conditions	Not approved by EMA
APALUTAMIDE	ERLEADA	Non-metastatic, castration-resistant prostate cancer	Approved by FDA in 2018
ARIPIPRAZOLE LAUROXIL	ARISTADA	Schizophrenia and related psychotic disorders	Not approved by EMA, orphan designation
ARTEMETHER; LUMEFANTRINE	COARTEM	Malaria in adults	Not approved by EMA, orphan designation
ASCORBIC ACID; POLYETHYLENE GLYCOL 3350; PO	PLENVU	Cleansing of the colon in preparation for colonoscopy	Not approved by EMA, orphan designation
AVATROMBOPAG MALEATE	DOPTELET	Thrombocytopenia in adult patients with chronic liver disease	Approved by FDA in 2018
BALOXAVIR MARBOXIL	XOFLUZA	Influenza A and B virus infection	Not approved by EMA
BELINOSTAT	BELEODAQ	Relapsed or refractory peripheral T-cell lymphoma	Not approved by EMA, orphan designation
BENZNIDAZOLE	BENZNIDAZOLE	Chagas disease in children	Not approved by EMA, orphan designation

BEPOTASTINE BESILATE	BEPREVE	Itchy eyes due to allergic conjunctivitis	Not approved by EMA
BESIFLOXACIN HYDROCHLORIDE	BESIVANCE	Bacterial conjunctivitis	Not approved by EMA
BETRIXABAN	BEVYXXA	Prophylaxis of venous thromboembolism	Not approved by EMA
CALASPARGASE PEGOL-MKNL	ASPARLAS	Acute lymphoblastic leukemia in pediatric and young adult patients.	Not approved by EMA
CANNABIDIOL	EPIDIOLEX	spasticity in adult patients with multiple sclerosis	Approved by FDA in 2018
CEMIPLIMAB-RWLC	LIBTAYO	locally advanced or metastatic cutaneous squamous cell carcinoma in patients not candidates for curative surgery or curative radiation	Approved by FDA in 2018
CHOLINE C-11	CHOLINE C-11	positron emission tomography imaging of patients with suspected prostate cancer recurrence and non-informative bone scintigraphy, computerized.tomography or magnetic resonance imaging	Not approved by EMA, cancer drug
CHOLINE FENOFIBRATE	TRILIPIX	Primary hypercholesterolemia or mixed dyslipidemia	Not approved by EMA
CITRIC ACID; MAGNESIUM OXIDE; SODIUM.PICOSU	PREPOPIK	Cleaning colon before colonoscopy	Not approved by EMA; withdrawn by the FDA
CONIVAPTAN HYDROCHLORIDE	VAPRISOL	Hypervolemic hyponatremia in hospitalized patients	Not approved by EMA
COPANLISIB DIHYDROCHLORIDE	ALIQOPA	Relapsed follicular lymphoma	Not approved by EMA, orphan designation
CRISABOROLE	EUCRISA	Mild to moderate atopic dermatitis	Not approved by EMA
CROFELEMER	MYTESI	Non-infectious .diarrhea in adult patients with HIV/AIDS	Not approved by EMA
DEFLAZACORT	EMFLAZA	Duchenne Muscular Dystrophy	Not approved by EMA, orphan designation
DELAFLOXACIN MEGLUMINE	BAXDELA	Acute bacterial skin and skin structure infections	Not approved by EMA
DEOXYCHOLIC ACID	KYBELLA	Appearance of moderate to severe fullness associated with submental fat in adults	Not approved by EMA
DESVENLAFAXINE SUCCINATE	PRISTIQ	Major depressive disorder in adults	Not approved by EMA

© 2022 Blankart K et al. *JAMA Network Open.*

DEUTETRABENAZINE	AUSTEDO	Chorea associated with Huntington's disease	Not approved by EMA
DIFLUPREDNATE	DUREZOL	Inflammation and pain associated with ocular surgery	Not approved by EMA
DORAVIRINE	PIFELTRO	HIV-1 infection in adult patients with no prior antiretroviral treatment history	Approved by FDA in 2018
DROXIDOPA	NORTHERA	Neurogenic orthostatic hypotension	Not approved by EMA, orphan designation
DUVELISIB	COPIKTRA	Relapsed or refractory chronic lymphocytic leukemia or small lymphocytic lymphoma after at least two prior therapies	Not approved by EMA, orphan designation
ECALLANTIDE	KALBITOR	Acute attacks of hereditary angioedema	Not approved by EMA, orphan designation
EDARAVONE	RADICAVA	neurological symptoms and damage from acute ischemic stroke and delaying disease progression of ALS	Not approved by EMA, orphan designation
EFINACONAZOLE	JUBLIA	Fungal infection of the nail	Not approved by EMA
ELAGOLIX SODIUM	ORILISSA	Management of moderate to severe pain associated with endometriosis	Not approved by EMA
ELAPEGADEMASE-LVLR	REVCOVI	Severe combined immune deficiency	Not approved by EMA, orphan designation
EMAPALUMAB-LZSG	GAMIFANT	Primary hemophagocytic lymphohistiocytosis	Not approved by EMA, orphan designation
ENASIDENIB MESYLATE	IDHIFA	Relapsed or refractory acute myeloid leukemia	Not approved by EMA, orphan designation
ERAVACYCLINE DIHYDROCHLORIDE	XERAVA	Complicated intra-abdominal infections	Approved by FDA in 2018
ESZOPICLONE	LUNESTA	Insomnia	Not approved by EMA
FILGRASTIM-AAFI	NIVESTYM	infection as manifested by febrile neutropenia, nonmyeloid malignancies	Not approved by EMA, orphan designation
FINAFLOXACIN	XTORO	Acute otitis erna (swimmer's ear)	Not approved by EMA
FLIBANSERIN	ADDYI	Hypoactive sexual desire disorder in premenopausal women	Not approved by EMA
FOSNETUPITANT CHLORIDE HYDROCHLORIDE; PALON	AKYNZEO	Prevention of acute and delayed nausea and vomiting, initial and repeat courses of moderately emetogenic cancer chemotherapy	Approved by FDA in 2018
FOSTAMATINIB DISODIUM	TAVALISSE	Chronic immune thrombocytopenia	Not approved by EMA, orphan designation
FREMANEZUMAB-VFRM	AJOVY	episodic and chronic migraine prevention and cluster headache	Approved by FDA in 2018
GALCANEZUMAB-GNLM	EMGALITY	Migraine prophylaxis and treatment of episodic cluster headaches	Approved by FDA in 2018

GALLIUM DOTATATE GA-68	NETSPOT	Preparation of gallium Ga 68 dotatate injection	Not approved by EMA, orphan designation
GILTERITINIB FUMARATE	XOSPATA	relapsed or refractory acute myeloid leukemia	Not approved by EMA, orphan designation
GLASDEGIB MALEATE	DAURISMO	Acute myeloid leukemia	Not approved by EMA, orphan designation
GLUCARPIDASE	VORAXAZE	Patients on methotrexate treatment who have kidney dysfunction	Not approved by EMA, orphan designation
IBALIZUMAB-UIYK	TROGARZO	HIV-1 infection in highly treatment-experienced adults	Not approved by EMA, orphan designation
IOBENGUANE SULFATE I-123	ADREVIEW	Detection of primary and metastatic pheochromocytoma or neuroblastoma	Not approved by EMA, orphan designation
IVOSIDENIB	TIBSOVO	Relapsed or refractory AML with a susceptible IDH1 mutation	Not approved by EMA, orphan designation
IXABEPILONE	IXEMPRA KIT	Various types of cancer	Not approved by EMA, cancer drug
LANADELUMAB (SHP643)	TAKHZYRO	Prevent attacks with hereditary angioedema	Approved by FDA in 2018
LAROTRECTINIB SULFATE	VITRAKVI	Solid tumors	Not approved by EMA, orphan designation
LATANOPROSTENE BUNOD	VYZULTA	Open-angle glaucoma or ocular hypertension	Not approved by EMA
LEVOLEUCOVORIN	KHAPZORY	Osteosarcoma	Not approved by EMA, orphan designation
LEVOMILNACIPRAN HYDROCHLORIDE	FETZIMA	Major depressive disorder	Not approved by EMA
LIFITEGRAST	XIIDRA	Signs and symptoms of keratoconjunctivitis sicca	Not approved by EMA
LOFEXIDINE HYDROCHLORIDE	LUCEMYRA	Acute withdrawal from opioids and for facilitation of the completion of opioid discontinuation treatment	Not approved by EMA
LORCASERIN HYDROCHLORIDE	BELVIQ	Obesity, adjunct to a reduced-calorie diet and increased physical activity	Not approved by EMA
LORLATINIB	LORBRENA	Metastatic non-small cell lung cancer	Approved by FDA in 2018
LUBIPROSTONE	AMITIZA	Chronic idiopathic constipation ;irritable bowel syndrome with constipation	Not approved by EMA
LUCINACTANT	SURFAXIN	Prevention of respiratory distress syndrome in premature infants	Not approved by EMA

© 2022 Blankart K et al. *JAMA Network Open.*

LULICONAZOLE	LUZU	Topical treatment of fungal infections	Not approved by EMA
LUSUTROMBOPAG	MULPLETA	Chronic liver disease	Approved by FDA in 2018
LUTETIUM DOTATATE LU-177	LUTATHERA	Somatostatin receptor-positive gastroenteropancreatic neuroendocrine tumors	Approved by FDA in 2018
MOGAMULIZUMAB-KPKC	POTELIGEO	Relapsed or refractory mycosis fungoides or Sézary syndrome	Approved by FDA in 2018
MOXETUMOMAB PASUDOTOX-TDFK	LUMOXITI	Relapsed or refractory hairy cell leukemia	Not approved by EMA, orphan designation
MOXIDECTIN	MOXIDECTIN	Onchocerciasis	Not approved by EMA, orphan designation
NALDEMEDINE TOSYLATE	SYMPROIC	Opioid-induced constipation	Not approved by EMA
NETARSUDI DIMESYLATE	RHOPRESSA	Open-angle glaucoma or ocular hypertension	Not approved by EMA
OBILTOXAXIMAB	ANTHIM	Anthrax exposure, bacterial infection, Crohn's disease, graft versus host disease	Not approved by EMA, orphan designation
OMACETAXINE MEPESUCCINATE	SYNRIBO	Accelerated or chronic phase CML	Not approved by EMA, orphan designation
OMADACYCLINE TOSYLATE	NUZYRA	Community acquired bacterial pneumonia; acute bacterial skin and skin structure infections	Not approved by EMA
OMEGA-3-ACID ETHYL ESTERS	LOVAZA	Severe hypertriglyceridemia	Not approved by EMA
OZENOXACIN	XEPI	Impetigo	Not approved by EMA
PEGINESATIDE ACETATE	OMONTYS	anemia due to chronic kidney disease	Not approved by EMA
PEGVALIASE-PQPZ	PALYNZIQ	Uncontrolled blood phenylalanine concentrations	Approved by FDA in 2018
PIMAVANSERIN TARTRATE	NUPLAZID	Neurologic disorders, parkinson's disease, psychosis, schizophrenia, schizoaffective and, sleep disorders	Not approved by EMA
PLAZOMICIN SULFATE	ZEMDRI	Complicated Urinary Tract Infections	Not approved by EMA
PLECANATIDE	TRULANCE	Stimulation of intestinal fluid secretions in the gastrointestinal tract to support regular bowel function	Not approved by EMA
POLIDOCANOL	ASCLERA	Uncomplicated spider veins and uncomplicated reticular veins in the lower extremity	Not approved by EMA
PRAMLINTIDE ACETATE	SYMLIN	Type 1 and type 2 diabetes mellitus as an adjunct to preprandial insulin	Not approved by EMA
RAMELTEON	ROZEREM	Insomnia characterized by difficulty with sleep onset	Not approved by EMA

RAVULIZUMAB-CWVZ	ULTOMIRIS	Paroxysmal nocturnal hemoglobinuria	Approved by FDA in 2018
RAXIBACUMAB	RAXIBACUMAB	(prophylaxis of) inhalational anthrax	Not approved by EMA, orphan designation
REVEFENACIN	YUPELRI	Chronic obstructive pulmonary disease	Not approved by EMA
RIFAMYCIN	AEMCOLO	Travelers' diarrhea, noninvasive strains of E. coli	Not approved by EMA
SACROSIDASE	SUCRAID	Congenital sucrose-isomaltase deficiency	Not approved by EMA, orphan designation
SARECYCLINE HYDROCHLORIDE	SEYSARA	Inflammatory lesions of non-nodular moderate to severe acne vulgaris	Not approved by EMA
SECNIDAZOLE	SOLOSEC	Bacterial vaginosis in adult women	Not approved by EMA
SODIUM ZIRCONIUM CYCLOSILICATE	LOKELMA	Hyperkalemia	Approved by FDA in 2018
SPINOSAD	NATROBA	Head lice	Not approved by EMA
SUVOREXANT	BELSOMRA	Insomnia characterized by difficulties with sleep onset	Not approved by EMA
TAFENOQUINE SUCCINATE	KRINTAFEL	Relapse of Vivax malaria	Not approved by EMA, orphan designation
TAGRAXOFUSP-ERZS	ELZONRIS	Blastic plasmacytoid dendritic cell neoplasm	Not approved by EMA, orphan designation
TALAZOPARIB TOSYLATE	TALZENNA	Deleterious or suspected deleterious germline BRCA mutated, HER2 negative locally advanced or metastatic breast cancer in adults	Approved by FDA in 2018
TAVABOROLE	KERYDIN	Onychomycosis (fungal infection)	Not approved by EMA
TECOVIRIMAT	TPOXX	Human smallpox disease	Not approved by EMA
TESAMORELIN ACETATE	EGRIFTA	Reduction of excess abdominal fat, HIV-infected patients with lipodystrophy	Not approved by EMA
TINIDAZOLE	TINDAMAX	Trichomoniasis caused by T vaginalis	Not approved by EMA, orphan designation
URIDINE TRIACETATE	XURIDEN	Hereditary orotic aciduria	Not approved by EMA, orphan designation
VALBENAZINE TOSYLATE	INGREZZA	Tardive dyskinesia	Not approved by EMA
VILAZODONE HYDROCHLORIDE	VIIBRYD	Major depressive disorder	Not approved by EMA
VORINOSTAT	ZOLINZA	Progressive, persistent or recurrent cutaneous T-cell lymphoma	Not approved by EMA, orphan designation

© 2022 Blankart K et al. *JAMA Network Open.*

ASPARAGINASE ERWINIA CHRYSANTHEMI	ERWINAZE	Acute lymphoblastic leukemia	Not approved by EMA, orphan designation
**Panel C: Medicines available in U.S. and subsequently withdrawn**			
ADALIMUMAB-ADBM	CYLTEZO	Multiple purposes: rheumatoid arthritis, psoriatic arthritis, Crohn's disease	Withdrawn / refused / suspended by EMA
ETEPLIRSEN	EXONDYS 51	Duchenne muscular dystrophy	Withdrawn / refused / suspended by EMA
ILOPERIDONE	FANAPT	acute schizophrenia	Withdrawn / refused / suspended by EMA
MIPOMERSEN SODIUM	KYNAMRO	homozygous familial hypercholesterolemia	Withdrawn / refused / suspended by EMA
PRALATREXATE	FOLOTYN	relapsed or refractory peripheral T-cell.lymphoma	Withdrawn / refused / suspended by EMA
ROMIDEPSIN	ISTODAX	cutaneous T-cell lymphoma or/and peripheral T-cell lymphoma	Withdrawn / refused / suspended by EMA
TALIGLUCERASE ALFA	ELELYSO	Type 1 Gaucher disease	Withdrawn / refused / suspended by EMA
**Panel D: Medicines available in U.S. and with German equivalents**			
BRIGATINIB	ALUNBRIG	Anaplastic lymphoma kinase positive, metastatic non-small cell lung cancer	Became available outside study period
MACIMORELIN ACETATE	MACRILEN	adult growth hormone deficiency	Became available outside study period

Notes: EMA: European Medicines Agency; FDA: Food and Drug Administration

© 2022 Blankart K et al. *JAMA Network Open.*
